# Verbal Information Transfer in Real-Life: When Mothers Worry About Their Child Starting School

**DOI:** 10.1007/s10826-017-0735-3

**Published:** 2017-05-06

**Authors:** Laura Pass, Kiki Mastroyannopoulou, Sian Coker, Lynne Murray, Helen Dodd

**Affiliations:** 1 Department of Clinical Psychology, University of East Anglia, Norwich, UK; 2 School of Psychology and Clinical Language Sciences, University of Reading, Reading, UK; 3 Department of Psychology, Stellenbosch University, Stellenbosch, South Africa

**Keywords:** Information transfer, Children, Parents, Anxiety, School transition

## Abstract

Verbal information transfer, one of Rachman’s three pathways to fear, may be one way in which vulnerability for anxiety may be transmitted from parents to children. A community sample of mothers and their preschool-aged children (*N* = 65) completed observational tasks relating to the child starting school. Mothers were asked to tell their child about social aspects of school; then children completed a brief play assessment involving ambiguous, school-based social scenarios. Mothers completed self-report questionnaires on social anxiety symptoms, general anxiety and depressive symptoms as well as a questionnaire on child anxiety symptoms and indicated whether they were personally worried about their child starting school. There was a significant difference in the information given to children about school between mothers who stated they were worried and those who stated they were not, with mothers who were worried more likely to mention unresolved threat, use at least one anxiety-related word, and show clear/consistent negativity (all *p*s < .01). Significant associations were also found between the emotional tone of mothers’ descriptions of school and children’s own representations of school. These findings support the theory that the information mothers give to their child may be influenced by their own concerns regarding their child, and that this verbal information affects child representations.

## Introduction

Anxiety is a normal human emotion. It is evolutionarily adaptive in alerting us and facilitating our response to potential threats in our environment (Bar-Haim et al. 2007). While fears in childhood are normal, following a developmental course relating to cognitive stages and capacities (Muris et al. 2000a), a substantial minority of children experience clinically significant levels of anxiety (Muris et al. 2000b). In fact, anxiety disorders are among the most common psychological difficulties in children (Polanczyk et al. 2015) and are associated with ongoing mental health problems into adulthood (Bittner et al. 2007), as well as poorer academic outcomes (Van Ameringen et al. 2003). There is convincing evidence that pathways to anxiety disorders can begin early in life (Spence et al. 2001). It is therefore important to understand how anxiety develops with a view to informing prevention and early intervention strategies.

It has consistently been shown that anxiety disorders run in families (Rapee and Spence 2004). In ‘bottom-up’ studies, parents of anxious children have been shown to be more likely to have an anxiety disorder than parents of non-anxious children (Cooper et al. 2006). Similarly, in ‘top-down’ studies, children of parents with an anxiety disorder are up to seven times more likely to have an anxiety disorder than children of control parents (Turner et al. 1987). While there is a substantial genetic component to the vulnerability to, and familial aggregation of, anxious temperamental traits (Gregory and Eley 2007) environmental factors are also important.

Rachman (1977, 1991) proposed that fear acquisition may occur via three pathways: direct conditioning, vicarious exposures (modelling/observational learning), and information transmission, or transfer. All three routes have received support in relation to the development of anxiety (Askew and Field 2008; King et al. 1998; Muris and Field 2010). The majority of studies investigating the information transfer pathway have focused on fear of animals, using the paradigm developed by Field (e.g., Field and Lawson 2003). Within this paradigm, children are typically provided with information about novel animals by an unfamiliar person or via written materials, and the effect of this information is then evaluated. Using this approach, studies have shown that negative (threat) information increases child fear beliefs, behavioural avoidance, and negative implicit attitudes toward the novel stimuli (Field et al. 2001, 2008; Field and Lawson 2003).

Recent models of parental influences on anxiety risk identify information transfer as one of the mechanisms by which parents might affect their child’s risk for anxiety (Creswell et al. 2011; Murray et al. 2009). To date, however, only a small number of studies have assessed the role of parents (mostly with mothers, who are most often in the primary caregiver role) in information transfer (for a full review see Percy et al. 2015). Using experimental designs, Muris’ research team have replicated the verbal information transfer effect described above within the context of parent–child interactions: parents who are given negative information about a novel animal transmit more negative information to their child than parents who are given positive information (Muris et al. 2010; Remmerswaal et al. 2010). This occurs even when parents are not explicitly instructed to pass on information to their child (Remmerswaal et al. 2013). Furthermore, when ambiguous information about a novel animal is given to parents (Muris et al. 2010), high trait anxious parents make more negative statements about the novel animal in their descriptions than low trait anxious parents. This, in-turn, corresponds to their child’s subsequent fear beliefs regarding the novel animal. Taken together, these studies clearly indicate that information transfer does take place between parents and their children, at least in laboratory settings and in relation to novel stimuli.

Investigating information transfer in a more naturalistic manner can be challenging, due to the greater variation in information and topics discussed compared to an experimental context using novel animals. Capitalising on a naturally occurring novel event can be one way to investigate this, such as the reaction to the swine flu epidemic investigated by Remmerswaal and Muris (2011). In this study, parental information transfer regarding swine flu fear was found to partially mediate the effect of parental swine flu fear on child swine flu fear. This study provides initial evidence to support the relevance of the information transfer pathway in a real-world context. However, only minimal data on information transfer was collected (a 4-item questionnaire) and the study was reliant on retrospective report so only limited conclusions can be drawn.

A further limitation of the current literature is that most studies of parent–child information transfer have focused on middle childhood. Parental information transfer may be particularly salient in early childhood, given the relatively limited sources of information to which young children are exposed. One study that explored such factors in early childhood was that of Murray et al. (2014). As part of a prospective longitudinal study (see Murray et al. 2007, 2008), information transfer by mothers with social anxiety disorder was compared to that of non-anxious mothers, using starting school as a disorder-relevant context. Around 3 months prior to starting primary school, mothers and children co-constructed a narrative about the child’s first day at school using a picture book prompt. A comprehensive coding scheme was developed to capture specific aspects of verbal information transmission of relevance to social anxiety. Mothers with social anxiety showed a significantly greater overall anxiogenic style in their narratives compared to non-anxious mothers; including being more likely to make a high (top 30% of scores) proportion of threat attributions and child vulnerability comments, and to fail to resolve their child’s expressed anxiety (where the mother ignored the expression of child concern either by not responding, or by providing an irrelevant response). Mothers with social anxiety also showed less positive encouragement in their narratives compared to control mothers. Children also completed a play representation of school based ambiguous scenarios, and maternal lack of positive encouragement in the narrative task was significantly associated with highly anxious/negative child representations (Pass 2010; Pass et al. 2012).

Assessing the impact of the information given is a considerable challenge with young children, who may be too immature to give adequate verbal accounts of their experiences. Accordingly, in order to capture anxiety-related child cognitions and beliefs in a developmentally appropriate way, a small number of studies have used doll play methodology (Dodd et al. 2011; Ooi et al. 2015; Pass et al. 2012; Warren et al. 2000). Doll play representations allow young children to readily enact their experience (Woolgar 1999), and have previously been used to investigate attachment related themes (e.g. the MacArthur Story Stem Battery; MSSB, Bretherton and Oppenheim 2003) and social representations in the context of maternal depression (Murray et al. 1999).

There is evidence from these studies that children’s representations using doll-play have predictive validity, including in relation to anxiety. For example, Warren et al. (2000) found that overall negative expectations, as represented in child doll play at 5 years of age, predicted parent/teacher reported child anxiety 1 year later. Furthermore, Dodd et al. (2011) found that threat interpretations of ambiguous scenarios, as assessed using doll-play at age 4, were associated with current anxiety diagnoses, and predicted anxiety symptoms at 12-month (although not at 2- or 5- year) follow up. The authors concluded that threat interpretations may play a role in the maintenance of child anxiety, but not necessarily the initial development of anxiety symptoms. Finally, Pass et al. (2012) found that children of mothers with social anxiety were more likely to provide anxious-negative responses in their doll play, and that this predicted teacher-reported anxious/depressed symptoms and social worries at the end of their first term at school.

One recent study has used the story-stem methodology to examine the information transfer pathway in parents and preschool children. Ooi et al. (2015) asked parents to provide a written ending to a set of story stems as if they were telling the story to their child and explored the association between these parental story endings and the child’s own endings, or interpretations, assessed independently using doll play. The authors found that threat in parental written story stem responses was associated with child negative interpretation bias on these same stories. As parents were not recorded actually passing this information on to their child, the extent to which the written responses accurately reflect how they would verbally transmit this information is unknown.

Starting primary school is a significant developmental step in early childhood (Dockett and Perry 1999a), and is a potentially anxiety provoking, yet normative event for both children and parents. Both parents and child educators rate adjustment to the social aspects of school as the most important factor for a child’s transition (Dockett and Perry 1999a). By making use of this real-life social context, it is possible to explore whether mothers’ concerns about this specific and largely social challenge affect how they talk to their child about the topic, and how children represent such challenges. Previous work (e.g. Murray et al. 2014; Pass et al. 2012) has provided valuable insights into information transfer between parents and their children on this topic. However, the coding schemes for both maternal narratives and child play representations in these studies were very complex; each required a high level of training to become reliable and the time taken to train and code in such detail is not feasible in many settings.

**Fig. 1:**
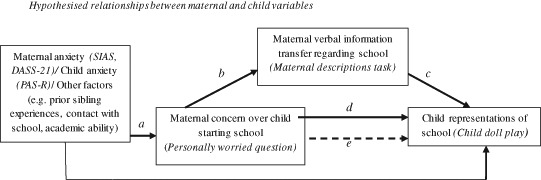
Hypothesised relationships between maternal and child variables *NB*: *SIAS* Social Interaction Anxiety Scale, *DASS-21* Depression Anxiety and Stress Scale, 21 item version; *PAS-R* Preschool Anxiety Scale Revised

The present study had two aims: To investigate information transmission in a sample of mothers and young children from the general population using the naturalistic context of starting primary school; and to develop a streamlined and engaging, clinician-friendly research tool that can be used in a community setting. Figure 1 depicts hypothetical associations between maternal and child factors based on the theory and experimental literature outlined. We have also included additional factors that we acknowledge may be associated with child representations, but that are beyond the focus of the present research (e.g. academic ability). It was hypothesised that: (i) Mothers with anxious symptoms, and mothers of children with anxious symptoms would be more likely to be worried about their child starting school (relationship *a* in Fig. 1); (ii) that mothers who are worried about their child starting school would convey more negative information about school to their child (relationship *b* in Fig. 1); (iii) that the information mothers give about school would be associated with children’s representations of school (relationship *c* in Fig. 1); (iv) that the children of mothers who are concerned about them starting school are more likely to represent school in a negative way (relationship *d* in Fig. 1). A potential mediational relationship (the effect of maternal concern on child representations would be mediated by maternal verbal information transfer) was also hypothesised, if all of the above were supported (relationship *e* in Fig. 1).

## Method

### Participants

Sixty five mothers and their preschool age children participated in the study. Families were included if the mother was the primary caregiver, and the child was due to start primary school in the next academic year. Families were recruited across East Anglia from local preschools, nurseries, playgroups and church play schemes as well as SureStart initiatives. Mothers who took part were also invited to pass on the study details to other mothers they knew who may be interested, to maximize recruitment. Informed consent was obtained from all individual participants included in the study. The sample comprised white (100%) generally well-educated mothers (mean age when left full-time education = 21.20, SD = 3.55), most of whom were cohabiting with the child’s father (90.8%). Of the children, almost all were white (96.9%), 64.6% were female, and 63.1% were either first-born children or an only child (i.e. were the first child in their family to make the transition to school). The mean age of mothers at time of assessment was 37.23 years (SD = 4.72), while the mean child age was 50.72 months (SD = 4.09). None of the children had a significant physical illness or medical condition (which may have meant their transition to school was not typical), and all mothers and children spoke fluent English. Ethical approval for the study was obtained from the University of East Anglia Research Ethics Committee (ref: 2011/2012-47).

### Procedure

Families were sent information about the study via preschools and nurseries, and interested mothers contacted the researcher for more information and returned signed consent forms. All assessments subsequently took place in the family home, in the months preceding the child starting school. The ‘time lag’ in terms of number of days between the assessment and school start date was calculated (range 13–191 days, mean = 95 days). A typical school start date was used for this calculation as not all mothers knew their child’s exact start date at the time of taking part. The researcher gave a brief overview and gained verbal assent from the child, before completing a warm-up task with them (colouring in a school picture). Mothers were then asked to complete the school descriptions task with their child (speaking directly to them in the naturalistic setting of their own home), after which they completed the questionnaires while the researcher undertook the doll play task with the child.

For the doll-play task, the researcher introduced the child to the dolls and arranged the scenes for each scenario (coloured sheets of paper with drawings to represent each scenario, other dolls, table and chair props as appropriate). The child was encouraged to identify one doll to represent him/herself, to increase engagement and validity of the school context. The child was then asked to finish stories that the researcher started. The researcher followed a script to introduce each scenario, before asking for responses from the child to create endings for each. After each scenario the child was given a sticker to put on their school picture. After the doll play task, questionnaires were collected and the family was given an opportunity to ask questions. The child was given their school picture to keep and a larger sticker to thank them for their help, and mark the end of the visit.

### Measures

#### Maternal anxiety symptoms

##### Social interaction anxiety scale (SIAS; Mattick and Clarke 1998)

The SIAS is a 20-item questionnaire assessing worry relating to social interaction (e.g., ‘When mixing socially I am uncomfortable’), a key component in social anxiety. Each question is rated on a 5 point Likert scale, from 0 (Not at all characteristic or true of me) to 4 (Extremely characteristic or true of me). Total scores range from 0–80, with higher scores indicating greater social anxiety. The SIAS shows high internal consistency (α range .88–.94 across a variety of samples), test-retest reliability (r = .92 across both a 4-week and 12-week interval), and discriminates between clinical anxiety groups and between social anxiety and general population samples (Mattick and Clarke 1998). Internal consistency in this sample was good (α = .75).

##### Depression anxiety stress scales (DASS; Lovibond and Lovibond 1995)

The 21-item version of the DASS was used to measure general anxiety, stress and depressive symptoms. The scale shows high internal consistency on each of the three subscales (α range .82–.91), adequate concurrent validity between the subscales and related measures of anxiety/depression (r range .68–.85), and good test re-test reliability (Brown et al. 1997). Each subscale showed good internal consistency in this sample (α range .86–.87).

##### Maternal anxiety about their child starting school

In addition to the symptom questionnaires, a specific question was added: ‘Do you personally feel worried about your child starting school ? Yes/No’ ? This was to identify specific concerns mothers may have around their child starting school, which was not captured by any of the other questionnaires.

#### Child anxiety symptoms

##### The preschool anxiety scale- revised (PAS-R: Edwards et al. 2010)

The PAS-R is a revised version of the original PAS (Spence et al. 2001), and is a 28-item questionnaire designed for parents of children aged 2.5–6 years old, to assess child anxiety. The PAS-R shows strong internal consistency (total scale α = .92, subscales α = .72–.83), and construct validity (Edwards et al. 2010), including acceptable internal consistency in this sample (total scale α = .89, subscales α = .69–.83).

#### Maternal description of school task

The maternal description task was designed specifically for this study. Mothers were asked ‘Please tell your child something you think it is important for them to know about: (i) Making friends at school; (ii) Teachers at school; (iii) Playtime at school; and (iv) Older children at school’. Mothers were asked to spend up to 1 minute talking about each topic. This was completed in a naturalistic way, where the mother was seated with their child and spoke directly to them about the topics (i.e. mothers were recorded actually talking with their child, unlike some previous studies e.g. Ooi et al. 2015). The researcher provided the above prompts on a written sheet and was present during the task, but did not provide further input. It took mothers on average 5 minutes to complete the task.

The maternal descriptions task was audio recorded, and a coding scheme was developed to code positive and negative information. The coding scheme was based on previous coding schemes for capturing parental information transfer (e.g., Muris et al. 2010; Murray et al. 2014; Pella 2011) and theoretical/empirical models of transmission of anxiety (Field et al. 2001; Murray et al. 2009; Wood et al. 2003). A key aim for information transfer coding was that this was possible to complete directly from audio recordings, without the need to transcribe and separate comments into individual utterances (as for the more comprehensive coding scheme used by Murray et al. 2014; Pella 2011). This would allow the coding scheme to be used by both researchers and clinicians with limited time and resources. Seven maternal description codes were created: (i) Presence of unresolved threat (e.g., ‘Are you scared of the older children?’ without a follow-up statement indicating a way the child could cope, or a positive outcome or reassurance; (ii) positive encouragement (e.g., ‘You will have lots of new toys to play with’); (iii) use of an anxiety-related word (e.g., ‘shy’); (iv) negative evaluation of child (e.g., ‘What if the teacher tells you off because you’re naughty?’) (v) positive evaluation of child (e.g., ‘Your teacher will think you’re really helpful’); and (vi) overall negativity (none/minimal vs. clear/consistent) and (vii) overall positivity (none/minimal vs. clear/consistent), which were scores given to capture the overall positive/negative tone of what the mother had said.

Threat was coded only if this was unresolved (e.g. ‘You might not know where to go on your first day’ = unresolved threat). If a potential threat was voiced but then the mother provided a resolution, this was not coded as threat information. This was because there was often factual information provided within a positive frame (e.g. ‘you might not know where to go on your first day…but the older children will help you find your class’, or ‘…but that’s ok, it will be lots of other children’s first day too’). Due to the brief nature of the descriptions provided by mothers, and the fact that mothers tended to combine the different school topics in their speech, each code was rated across the entire maternal description rather than for each individual topic. Again, to streamline coding (and due to mothers combining topics) each code was rated as present/absent across the entire description of school (for the global codes of overall negativity and positivity, these were coded as none/low vs. clear/consistent to focus on the clear and pervasive presence of these emotional tones). Inter-rater reliability was established on 27% of the sample for the finalised coding scheme (ICC/kappas range .77–1.) and the main coding was completed by an independent post-graduate researcher, who was blind to maternal and child anxiety symptoms, as well as child doll play.

#### Doll play (DP) task

The doll play task comprised three ambiguous scenarios, presented using dolls and simple props. A researcher presented the scenarios using a story-stem approach (e.g. MacArthur Story Stem Battery: MSSB; Bretherton and Oppenheim 2003) where the beginning of a scenario was set up by the researcher, but the child was left to end each story. All three scenarios focused on social situations that may occur in reality at school (seeing older children look over at you, being told the teacher is looking for you, hearing other children laughing in the playground, see Appendix A), and were informed by previous work (Barrett et al. 1996; Pass 2010; Pass et al. 2012). In line with the maternal descriptions coding scheme, a key aim for the child representations coding scheme was that it was concise, and coding could be completed directly from videos to increase the applicability of the tool for clinical use. Therefore, the entire doll play was coded for overall positivity and overall negativity (both rated as none/minimal vs. clear/consistent, again to highlight the clear and pervasive presence of these emotional tones). Clear/consistent positivity was coded if there was more than one very positive representation within a scenario (e.g. another child asking them to play, then having fun together), or positivity across more than one scenario. Negativity was coded similarly, so clear/consistent negativity was coded if one scenario was extremely negative (e.g. teacher telling them off, child cries, parent has to come collect them and take them home) or negativity was present across more than one scenario (e.g. older children making fun of them, playground children do not want to play with them). A final ‘fun time at school’ scenario was included to ensure the play ended on a positive note, but was not coded. Inter-rater reliability was established on 27% of the sample (ICC/kappas range .72–1) by a separate post-graduate researcher who was experienced in coding doll play assessments, and was blind to all maternal data. The doll play task took around 15–20 minutes to complete.

### Data Analyses

None of the continuous variables were normally distributed, with the exception of the PAS-R, which met the assumptions for parametric testing. The remaining variables were categorical and largely dichotomous. Therefore bootstrapped correlations (Pearson’s for two continuous variables and point-biserial for one continuous and one categorical variable) were used, along with phi correlations for two categorical variables.

The potential influence of demographic variables on maternal questionnaires, description variables and doll play variables was assessed. No significant correlations emerged (*p* > .1), with two exceptions: a significant positive correlation between negative evaluation in maternal descriptions and time lag (*r* = .123, [.008, .250]), and a significant phi correlation between gender and overall positivity (phi = −.246, *p* = .048), where boys were more likely to show no/minimal positivity in their representation. These demographic variables were controlled for in all further analyses involving these two variables. For clarity of presentation, however, simple correlations are presented as patterns of significance did not change after controlling for them.

As child anxiety could feasibly affect what mothers say to their children and/or the child’s representations, the effect of child anxiety was also considered with the option to control for this if it was significantly associated with these factors. Associations between PAS-R scores and maternal description variables, and doll play variables, were examined. None of these point-biserial correlations was significant (all *p*s > .1), therefore child anxiety was not controlled for in further analyses.

## Results

Initial analyses focussed on the association between maternal and child anxiety and maternal concerns over their child starting school (relationship *a* in Fig. 1). Secondly, the association between maternal concerns and maternal descriptions of school was examined (relationship *b* in Fig. 1). Thirdly, the association between maternal descriptions and child representations of school (relationship *c* in Fig. 1) was assessed. The final analyses examined the association between maternal concerns and child representations of school (relationship *d* in Fig. 1).

Overall, 14 mothers (21.5% of the sample) reported feeling personally worried about their child starting school. This variable was not significantly correlated with DASS-21 anxiety scores, DASS-21 depression scores or SIAS scores, but was significantly correlated with the child’s anxiety as measured using the PAS-R (*r* = .406, *p* < .01). Mothers who stated they were personally worried about their child starting school scored their children as having significantly higher anxiety symptoms (mean = 38.73, SD = 12.24) than mothers who stated they were not personally worried about their child starting school (mean = 24.33, SD = 13.84).

Descriptive statistics for maternal descriptions variables are shown in Table 1, while the phi correlations between the ‘Are you personally worried about your child starting school?’ question and maternal description variables are shown in Table 2. Mothers who stated they were personally worried about their child starting school were significantly more likely to include at least one unresolved threat comment and at least one anxiety-related word in their narrative than mothers who stated they were not personally worried about their child starting school. Mothers who stated they were worried were also significantly more likely to show clear/consistent negativity across their description of school as a whole, than mothers who stated they were not worried about their child starting school. Furthermore, mothers who were personally worried were somewhat more likely to include some suggestion of negative evaluation and less likely to include suggestion of positive evaluation, although neither reached statistical significance (see Table 2).

**Table 1:** Descriptive data for maternal description variables

Maternal description variable (*N* = 65)	N (%) Absent	N (%) Present
Any unresolved threat(s)	52 (80.0%)	13 (20.0%)
Positive general comment(s)	7 (10.8%)	58 (89.2%)
Any anxious word(s)	55 (84.6%)	10 (15.4%)
Negative evaluation by others	59 (90.8%)	6 (9.2%)
Positive evaluation by others	52 (80.0%)	13 (20.0%)
	N (%) None/minimal	N (%) Clear/consistent
Overall negativity	53 (81.5%)	12 (18.5%)
Overall positivity	6 (9.2%)	59 (90.8%)

**Table 2:** Correlations between personally worried question and maternal description variables

Maternal description variable	Personally worried: yes
Presence of unresolved threat	.393**
Absence of positive general comments	.059
Presence of anxiety related word(s)	.503**
Suggestion of positive evaluation	−.206^
Suggestion of negative evaluation	.221
Overall maternal negativity: clear/consistent	.329**
Overall maternal positivity: none/minimal	.091

*NB*: All 2-tailed *p*-values*, ^* = *p* < .1, * = *p* < .05, ** = *p* < .01

The descriptive details for doll play variables are shown in Table 3. Table 4 shows the associations between maternal descriptions and child representations. Children of mothers who showed high negativity in their descriptions were more likely to show high negativity in their doll play than children of mothers who were less negative, although this only reached trend level (Fisher’s exact = .280; *p* = .057). There was also a significant association between lack of positivity in maternal descriptions and high negativity in child representations (phi = .311, *p* < .01).

**Table 3:** Descriptive data for doll play variables

Doll play variable	N (%) None/minimal	N (%) Clear/consistent	Total N
Overall negativity	49 (75.4%)	16 (24.6%)	65
Overall positivity	44 (67.7%)	21 (32.3%)	65

**Table 4:** Correlations between maternal description variables and doll play variables

Variables	DP overall negativity (none/minimal (0) vs. clear/consistent (1))	DP overall positivity (none/minimal (0) vs. clear/consistent (1))
MD Presence of unresolved threat	−.018	−.016
MD Absence of positive general comments	.147	−.028
MD Presence of anxiety related word(s)	−.046	−.021
MD Suggestion of positive evaluation	.107	.016
MD Suggestion of negative evaluation	.065	.007
MD Overall maternal negativity (none/minimal (0) vs. clear/consistent (1))	.280^	−.074
MD Overall lack of maternal positivity (clear/consistent (0) vs. none/minimal (1))	.311*	.007

*NB: MD* mother description variable*, DP* doll play variable*. (0)* indicates category coded as 0 for analysis, *(1)* indicates category coded as 1 for analysis

All 2-tailed *p*-values, *^* = *p* < .1, * = *p* < .05

The association between the Personally Worried question and both overall negativity and overall positivity in the doll play was also examined. There was no significant association between maternal concern over their child starting school and doll play negativity (phi = .048, *p* > .1) or doll play positivity (phi = −.202, *p* > .1).

## Discussion

The aim of the present research was to replicate and extend findings from our previous research (Murray et al. 2014; Pass et al. 2012) and the experimental literature, by examining information transfer between mothers and their children using the context of transition to school and a streamlined task designed to be useful in clinical and community settings. The first hypothesis was that higher maternal anxiety and child anxiety would be associated with maternal concern over their child starting school (relationship *a* in Fig. 1). While maternal anxiety was not associated with maternal concern over their child starting school, maternally reported child anxiety was. This perhaps reflects mothers’ view that anxious children may find changes such as the transition to school more difficult. It may be that this, rather than maternal anxiety, drives mothers from the general population to feel concerned over the school transition.

The second, and central, hypothesis of the present study was that mothers who were concerned about their child starting school would convey more negative information about school to their child, than mothers who did not have such concerns (relationship *b* in Fig. 1). In general, this hypothesis was supported: when talking to their child about school, compared to mothers who stated they were not worried about their child starting school, mothers who were personally worried were more likely to mention threat without resolving the threat, use at least one anxiety related word, and show higher negativity in their school descriptions as a whole. Taken together, these results suggest that mothers who hold personal concerns about the forthcoming novel event communicate more negative information about the novel event to their child.

The third hypothesis was that the verbal information mothers provide to their children would be associated with their child’s representation of school (relationship *c* in Fig. 1). This hypothesis was partly supported: children of mothers who expressed high negativity in their descriptions were more likely to represent school in a highly negative manner. Maternal positivity also appeared to be important, with low maternal positivity in school descriptions associated with high child representational negativity about school.

The final hypothesis was that children of mothers who were concerned about them starting school would be more likely to represent school in a negative way, compared to children of mothers who were not concerned (relationship *d* in Fig. 1). This hypothesis was not supported: no association was found between maternal concern over their child starting school, and child representations of school. This lack of association between maternal concern and the child’s representations suggests that maternal concern does not directly affect their child’s representation of school. As such, the potential mediation model depicted in Fig. 1 (relationship *e*) is not supported.

Taken together, the findings provide support for the hypothesised pathway of information transfer from parent to child (Creswell et al. 2011; Murray et al. 2009), and the information transfer pathway to fear proposed by Rachman (1977, 1991). The results parallel those of experimental studies, which have shown that parents transmit the information they are given about novel stimuli to their child (Muris et al. 2010; Remmerswaal et al. 2010, 2013). Importantly, the present research demonstrates that information transfer occurs in a naturalistic setting and when the topic is a complex, diverse and normative event. The only studies to have previously attempted this relied on retrospective questionnaires (Remmerswaal and Muris 2011), or used an in-depth coding methodology that is beyond the scope of regular clinical research use (Murray et al. 2014; Pass et al. 2012).

The lack of association between maternal concern about the child starting school and the child’s representations of school was unexpected. It may be that some of the mothers have specific concerns about their child’s ability to cope with the more practical aspects of school (e.g. managing the longer day without a nap, being independent from their mother), or in fact their own ability to cope (e.g. worried about how they will feel when their child is at school full-time) when their child starts school. These may not have affected the child’s representations of school as measured in the present study because we focused on social aspects of school in the doll-play task, which may not align with the mothers’ central concerns. Additionally, the simple dichotomy of yes/no answer to the question of whether mothers were concerned about their child starting school may have obscured subtle differences, where a continuous scale may have yielded greater sensitivity to detect such effects.

Although child representations are not a direct measure of anxiety, the fact that associations were found between what mothers say about school, and how children represent the setting, suggests information transfer might play an important part in developing child expectations. Given that mothers were more likely to be concerned about their child starting school if their child was anxious, mothers may try to support their child by acknowledging the difficulties they might face (e.g. ‘you might feel a bit shy’). This may inadvertently lead the child to represent school as a threatening place if the parent includes *unresolved* threat (i.e. highlighting threats in the school environment without following this up with a statement about how the child might cope, or how the threat might be resolved by others) and higher overall negativity. Such information transfer in the context of already heightened child anxiety symptoms has the possibility to make the transition to school even more challenging, and enhance risk in a vulnerable child. If replicated, these findings provide important implications for developing practical information-giving strategies for parents who hold realistic concerns about their child’s school transition

### Strengths and Limitations

This study had a number of strengths, most notably the use of the transition to primary school as a naturalistic upcoming social challenge in which to assess information transfer. Only a few studies have used a naturalistic context to investigate information transfer, despite this being an established theoretical pathway to the development of anxiety disorders (Creswell et al. 2011; Murray et al. 2009; Rachman 1977, 1991). Experimental studies using artificial tasks can only provide limited information on how the process operates in real-life; the present study extends previous findings by replicating information transfer occurring in relation to a naturally occurring, real-life universal event and demonstrating this via observational methodology and streamlined coding systems. This greatly enhances the ecological validity of the findings and makes the task more appropriate for clinical and community use. For example, adults involved in early childhood development and school transition (e.g. health visitors, preschool workers, primary school teachers) could also use the method and coding scheme of the current study as a clinical tool to identify families where school transition may be of particular concern, and to develop interventions based on modelling positive verbal communication through play.

An additional strength is the focus on a young and restricted age-range. Despite the fact that parents are likely to have a strong influence on young children, limited research has examined anxiety-related information transfer in preschool-aged children. The small age range in the present study allows us to be confident that the current data indicate how mothers of children in their pre-school year talk to their child about starting school. Furthermore, the relationship between information transfer and child representations found in previous work involving mothers with clinically significant levels of anxiety (Murray et al. 2014; Pass 2010; Pass et al. 2012) was replicated, using swift, easy to administer coding schemes.

It is true that the use of present/absent codes prevented fine detail analysis of content (e.g., proportion of negative statements relative to non-negative statements in maternal descriptions). However, the streamlined coding method for both maternal descriptions and child representations enhances the clinical utility of the measures, which was also a strength of the current study. Certain weaknesses of the current study should also be acknowledged. The sample was self-selected from the general population, and included mainly non-anxious mothers who were generally fairly positive about their child starting school. More selective recruitment of mothers who were anxious about their child’s transition to school may have yielded more potent information transfer effects, therefore these results are likely to be a conservative estimate.

The sample was largely recruited from formal preschool day care providers, which meant all children in the sample had current experience of formal day care, so were likely to have greater understanding of the forthcoming school transition than children not in a structured day care setting. Nevertheless, given that almost all UK children in their pre-school year receive some formal care (92% in 2014–2015; Department of Education 2016) the current sample was not overly unrepresentative in this respect.

The demographic characteristics highlight the homogeneity of the sample, with the vast majority of mothers being highly educated, of White British ethnic background, and co-habiting with the participating child’s father. No fathers were included due to recruitment restrictions and to minimise variation, although this did prevent fathers who were primary caregivers being considered. The sample characteristics necessarily limit the generalisability of findings to the wider population of the UK and beyond. The selection of families from the general population was a strength of the present study, as this allowed the universal aspect of school transition to be considered, including specific concerns around this irrespective of clinical anxiety in mother or child.

In addition, the cross-sectional design meant that causation cannot be established from these correlational results. The parenting pathways to anxiety model (Creswell et al. 2011; Murray et al. 2009) proposes that parental negative information transfer increases the risk of the development of child anxiety. However, the bi-directional influences within parenting are also included in this model, and there is increasing research in this area (Pardini 2008). While alternative designs are better able to answer hypotheses as to the direction of effects, they also require much greater time and resources to conduct (e.g., via longitudinal studies) or lack ecological validity (e.g., with experimental studies). The present study provides a workable method for naturalistic information transfer research in a general community population.

The doll play task was administered only once, immediately following the maternal description of school. Other studies have used a repeated-measures design, assessing child interpretations, anxiety, fear or beliefs about the event/object before and after the information transfer task, which allows the influence of the information given to be carefully measured (e.g., Remmerswaal et al. 2013). A number of factors affected our decision to administer the doll-play task only once. First, we did not want to overburden the young participants with excessive tasks. Second, in pilot research we found that young children are very reluctant to repeat a story they have done previously. Third, the focus of the current study was not a specific, novel event/object, but rather a forthcoming social challenge for which families had been preparing children already. Therefore the observational tasks were designed to capture how mothers and children thought/talked about school in general, with the assumption that other similar discussions had already taken place. Nevertheless, due to this design, the extent to which the child’s representation of school in the doll-play task was influenced by the information their mother had just given to them, rather than reflecting their general representation of school, remains unclear.

Investigating information transfer in a naturalistic context can be challenging, but the current study provides an important step forward in developing appropriate tasks for this purpose. The current findings suggest that in a sample from the general population, maternal concern about a specific upcoming event, starting school, is associated with particular ways of communicating about this event to their child, and that this communication might affect child representations of school.

## Appendix A

**Doll play scenarios**

**Scenario 1** Older children: [set up: playground scene, participant doll on one side of playground, group of 3 bigger dolls on other side]

“You’re in the playground at big school (name), and you see a group of older children who are a bit bigger than you, who are also in the playground. They look over and say something you can’t quite hear. What do you think they are saying? How do you feel? Can you show me what you do? What do the other children do? How does the story end?”

**Scenario 2** Teacher: [set up: participant doll on chair at desk in classroom scene, teacher doll at front of class, other child doll at side]

“You are in the classroom, and busy at your desk working. You are busy doing a drawing in your class when someone comes to tell you the teacher is looking for you. Why is the teacher looking for you? Can you show me what you do? How does the story end?”

**Scenario 3** Playground game : [set up: Different playground scene, different similar sized dolls in playground moving around, participant doll moved closer towards them]

“You’ve done lots of things in school and now it’s time for your break. The bell rings and you go out in the playground. Some children from the other class who you don’t know are already there, and they are playing a great game that you really like (ask child to give example of a game they like to play, and use this). As you walk near them, you can hear the children laughing. Why are the children laughing? How do you feel when you’re near the children and you hear them laughing? Can you show me what you do? How does the story end? (If this doesn’t end positively, say: then one of your friends sees you and you play together and have fun).”
